# Exploring the Role of Surface and Mitochondrial ATP-Sensitive Potassium Channels in Cancer: From Cellular Functions to Therapeutic Potentials

**DOI:** 10.3390/ijms25042129

**Published:** 2024-02-09

**Authors:** Dong-Oh Moon

**Affiliations:** Department of Biology Education, Daegu University, 201, Daegudae-ro, Gyeongsan-si 38453, Gyeongsangbuk-do, Republic of Korea; domoon@daegu.ac.kr; Tel.: +82-53-850-6992

**Keywords:** KATP channel, cancer, mitochondria, potassium

## Abstract

ATP-sensitive potassium (KATP) channels are found in plasma membranes and mitochondria. These channels are a type of ion channel that is regulated by the intracellular concentration of adenosine triphosphate (ATP) and other nucleotides. In cell membranes, they play a crucial role in linking metabolic activity to electrical activity, especially in tissues like the heart and pancreas. In mitochondria, KATP channels are involved in protecting cells against ischemic damage and regulating mitochondrial function. This review delves into the role of KATP channels in cancer biology, underscoring their critical function. Notably responsive to changes in cellular metabolism, KATP channels link metabolic states to electrical activity, a feature that becomes particularly significant in cancer cells. These cells, characterized by uncontrolled growth, necessitate unique metabolic and signaling pathways, differing fundamentally from normal cells. Our review explores the intricate roles of KATP channels in influencing the metabolic and ionic balance within cancerous cells, detailing their structural and operational mechanisms. We highlight the channels’ impact on cancer cell survival, proliferation, and the potential of KATP channels as therapeutic targets in oncology. This includes the challenges in targeting these channels due to their widespread presence in various tissues and the need for personalized treatment strategies. By integrating molecular biology, physiology, and pharmacology perspectives, the review aims to enhance the understanding of cancer as a complex metabolic disease and to open new research and treatment avenues by focusing on KATP channels. This comprehensive approach provides valuable insights into the potential of KATP channels in developing innovative cancer treatments.

## 1. Introduction

KATP channels are crucial in controlling cell functions across various tissues such as the heart, pancreas, and brain. Their distinctive characteristic lies in their responsiveness to the cell’s metabolic condition, particularly to alterations in ATP/ADP ratios, thereby linking cellular metabolism with electrical activities [1,2]. Recent research has increasingly highlighted the important role of KATP channels in the field of oncology.

Cancer cells are known for their rampant growth and proliferation, necessitating unique metabolic processes and signaling pathways, unlike normal cells. KATP channels’ capacity to influence membrane potential and the pathways of intracellular signaling is vital in comprehending the physiology of cancer cells [3,4]. This review is focused on dissecting the complex roles of KATP channels within cancer cells, examining their impact on the distinct metabolic and ionic makeup of tumor environments.

Initially, we will investigate the fundamental architecture and operational mechanisms of KATP channels, emphasizing their regulatory functions. Subsequently, the focus will shift to their evolving significance in oncology, exploring how changes in their expression and functionality can affect cancer cell endurance, growth, and spread. The review will also discuss current studies on the potential of KATP channels as targets in cancer therapies, including the associated challenges and prospects.

Incorporating insights from molecular biology, physiology, and pharmacology, this thorough analysis aims to present an integrated perspective on the role of KATP channels in cancer. This contributes to the broader understanding of cancer as a multifaceted metabolic disorder. By elucidating the complex interactions between KATP channels and cancer cells, this review seeks to pave the way for new research and treatment approaches in combating cancer.

## 2. Biological Functions of Potassium

Potassium (K^+^) is an essential electrolyte that maintains cell membrane potential, regulates fluid balance, supports nerve and muscle function, including heart rhythm, and contributes to overall electrolyte and acid–base balance in the body. This review emphasizes the diverse and vital roles of K^+^ in cancer cell physiology, encompassing its impact on glycolysis, cell cycle progression, apoptosis, and metastasis.

K^+^ is crucial for regulating glycolysis in cancer cells, with hexokinase I and II overexpression initiating glucose metabolism critical for cancer cell growth [5,6]. The activity of hexokinase II, influenced by intracellular K^+^ levels affected by plasma membrane K^+^ channels such as Kv1.3, modulates glycolytic activity [7]. Pyruvate, the end product of glycolysis, either enters the TCA cycle or converts to lactic acid based on oxygen availability [8]. Pyruvate kinase, particularly its PKM2 isoform, acts as a metabolic switch in cancer cells, promoting lactic acid production. K^+^ and other monovalent cations’ influence on PK, including PKM2, highlights K^+^ channels’ significant role in cancer metabolism. 

K^+^ plays a pivotal role in the regulation of the cell cycle, being a key intracellular ion essential for maintaining the electrochemical gradient across cell membranes. During the G1 phase of the cell cycle, membrane hyperpolarization occurs, a process significantly influenced by growth factors like IGF-1. These growth factors interact with receptor tyrosine kinases (RTKs), such as IGF-1R, triggering pathways like extracellular signal-regulated kinase1/2 (ERK) and Phosphoinositide 3-kinase (PI_3_K). This activation leads to an increase in the expression and activity of various K^+^ channels, including Kv channels and calcium (Ca^2+^)-activated K^+^ channels. The resulting hyperpolarization of the membrane establishes an electrical gradient that promotes Ca^2+^ influx through channels such as Ca^2+^ release-activated Ca^2+^ (CRAC) channel1, CRAC3, transient receptor potential canonical 1 (TRPC1), and TRPV6. This influx of Ca^2+^ further regulates the activity and expression of Ca^2+^-activated K^+^ channels, sustaining the hyperpolarized state and enhancing Ca^2+^ entry. The elevation in intracellular Ca^2+^ levels activates Ca^2+^-dependent signaling enzymes, impacting the expression and activity of transcription factors like FOS, JUN, NFAT, and C-MYC. This sequence of events triggers the expression of cyclins and CDKs while inhibiting CDK inhibitor proteins, including p27KIP1 and p21waf1/cip1 [9,10,11]. Additionally, K^+^ is instrumental in controlling cell volume, which is crucial for cell cycle progression. For example, the Eag2 channels regulate the expression of cyclin B1 through the p38 MAP kinase pathway during the M phase of the cell cycle, demonstrating another dimension of K^+^’s role in cell cycle regulation [12].

K^+^ plays a crucial role in apoptosis by regulating enzymatic activities essential for cellular breakdown. High intracellular K^+^ levels inhibit enzymes like nucleases, necessary for DNA degradation during apoptosis, with inhibition occurring at normal cellular K^+^ concentrations (around 150 mM) [13]. K^+^ also affects caspase activation, where physiological levels of K+ inhibit apoptotic enzyme cascades. Apoptotic cells exhibit a significant K^+^ concentration reduction to about 35 mM, leading to enzyme activation involved in apoptosis [13]. This K^+^ efflux is associated with cell shrinkage, a characteristic of apoptosis, facilitating the activation of apoptosis-inducing enzymes [14]. Experiments involving K^+^ depletion and K^+^ channel overexpression have confirmed apoptosis induction across various cell types. 

In cancer cells, the modulation of K^+^ channels present a potential therapeutic target. The involvement of these channels in apoptosis varies according to cancer type, channel type, and their regulation by factors influencing cell death or survival. Overall, K^+^’s role in apoptosis, particularly through its effects on enzymatic activities and cell volume regulation, offers novel insight into the mechanisms of apoptosis regulation. This understanding has significant implications for developing therapeutic strategies targeting K^+^ channels in cancer treatment.

**Figure 1:**
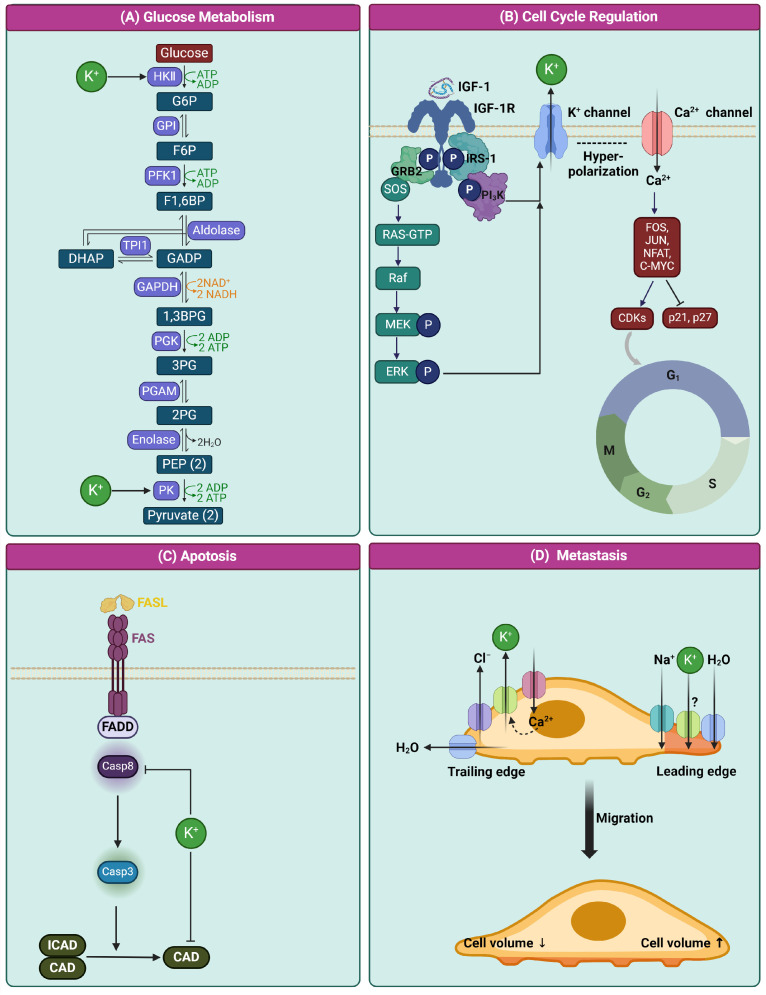
Schematic Representation of Potassium’s (K^+^) Role in Various Cellular Processes. (**A**) K^+^ interaction with hexokinase II (HKII) initiates glycolysis by converting glucose to glucose-6-phosphate (G6P). This segment traces the glycolytic pathway, showing the influence of K^+^ on key metabolic enzymes, including pyruvate kinase (PK), leading to pyruvate production and its metabolic outcomes in varying oxygen conditions. (**B**) The role of K^+^ in cell cycle regulation is depicted, beginning with the engagement of insulin-like growth factor 1 (IGF-1) with its receptor IGF-1R. Activation of PI_3_K/Akt and RAS/ERK pathways leads to K^+^ channel-mediated membrane hyperpolarization, affecting Ca^2+^ uptake and activating transcription factors that govern cell cycle progression. (**C**) K^+^ is depicted as an inhibitor of apoptosis, where elevated K^+^ levels suppress the activation of caspase-8 (Casp8) and the CAD enzyme, highlighting its role in inhibiting the apoptotic process. (**D**) K^+^’s control over cell volume changes during cancer cell migration is shown, with a focus on the polarized distribution of K^+^ channels and other ion channels at the cellular edges. The coordination of these channels is critical for facilitating cell migration, a vital process in cancer metastasis. The question mark indicates that the exact potassium channel is not known. Cartoon in Figure 1 was created with BioRender.com.

In the context of cancer metastasis, the role of K^+^ is critically linked to its influence on cell migration and invasion, which are key processes in the metastatic cascade. This aspect of cancer progression is not only crucial but often determines the lethality of the disease. Metastasis is responsible for over 90% of cancer-related deaths, with cell migration playing a central role in this process by enabling the spread of primary tumor cells to distant sites in the body [15]. The migration of cancer cells is characterized by dynamic changes in cell volume, which are crucial for the movement of cells. The leading edge of a migratory cell extends by increasing its volume, which is facilitated by the influx of ions and water. K^+^ channels play a role in this process by helping to regulate the cell volume at both the leading and trailing edges of the cell. During cell migration, K^+^ channels exhibit a polarized subcellular distribution. This distribution is essential for the localized hydrodynamic changes in the cell. At the cell’s trailing edge, Ca^2+^ entry activates Ca^2+^-activated K^+^ channels, leading to K^+^ efflux [16]. This efflux is part of the mechanism that reduces cell volume at the trailing edge, a critical step for cell motility. K^+^ channels work in coordination with Cl- channels, Na^+^ channels, and Na^+^-K^+^-Cl^-^ cotransporters to maintain ionic and water homeostasis during cell migration. This coordinated activity is crucial for the local changes in cell volume that drive the protrusion and retraction of the cell’s leading and trailing edges, respectively. The precise function of certain K^+^ channels, such as Kir4.2, situated at the leading edge of migrating cells [17], remains a subject of ongoing research. However, it is hypothesized that K^+^ influx through Kir channels at the leading edge could occur due to a local reversal of the K^+^ electrochemical gradient. The direction of K^+^ flow through these channels, whether inward or outward, depends on the local electrochemical gradient and is crucial for understanding their precise role in cell migration and metastasis. The movement of cancer cells through tissues requires the degradation of extracellular matrix proteins. K^+^ channels, by regulating the cell volume and maintaining the ionic balance, indirectly facilitate these processes, aiding in tumor cell invasion. Figure 1 depicts the physiological role of K^+^ in various cellular processes. 

The functions of K^+^ as outlined above are mediated by a variety of K^+^ channels. K^+^ channels are primarily classified into four distinct groups based on their structural characteristics and functional roles: voltage-gated (Kv), calcium-activated (KCa), inwardly rectifying (Kir), and two-pore-domain (K2P) potassium channels. Within the inner mitochondrial membrane, several specialized types have been identified, including mitochondrial KATP channels, mitochondrial large-conductance calcium-activated potassium (mitoBKCa) channels, mitochondrial voltage-dependent potassium (mitoKv1.3) channels, and twin-pore TASK-3 potassium channels. The discovery of the mitochondrial KATP channel was first made in liver mitochondria, with subsequent findings in various tissues such as the heart, brain, kidney, skeletal muscle, human T lymphocytes, and even amoeba mitochondria. This article will focus on the significance of KATP channels within cancer cells, underscoring their distinct functionality among the diverse potassium channel types. 

## 3. Structure and Regulation of KATP Channels

Plasma membrane KATP channels play a pivotal role in various tissues, acting as key mediators between cellular metabolic activity and membrane excitability. These channels are formed as hetero-octameric complexes, composed of four inward rectifier K^+^ channel subunits (Kir6.1 or Kir6.2, corresponding to genes KCNJ8 and KCNJ11, respectively) and four sulfonylurea receptor subunits (SUR1 or SUR2, associated with genes ABCC8 and ABCC9, respectively). The process of alternative splicing of ABCC9 gives rise to unique SUR2A and SUR2B subunits, which differ in their carboxyl terminal amino acid sequences. The spatial relationship of KCNJ8 with ABCC9 on chromosome 12 and of KCNJ11 with ABCC8 on chromosome 11 indicates a likely co-regulation and gene duplication phenomenon [18]. Each Kir6 unit encompasses two membrane-spanning domains surrounding a pore loop, crucial for K^+^ selectivity. These units feature a significant cytoplasmic domain at the amino and carboxyl ends. The SUR subunits, part of the ATP-binding cassette superfamily, consist of 17 transmembrane domains along with two intracellular nucleotide-binding folds. These elements together establish two adenine nucleotide-binding sites, ABS1 and ABS2, at their interface [19,20]. 

**Figure 2:**
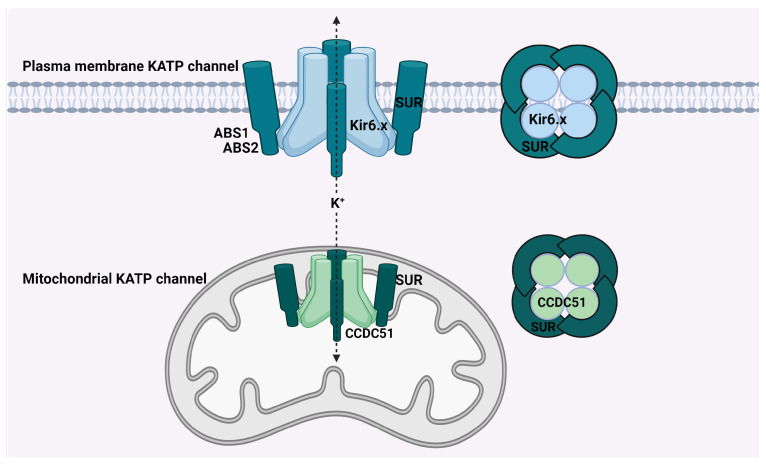
Structural Comparison of Plasma Membrane and Mitochondrial KATP Channels. The plasma membrane KATP channel is depicted as a hetero-octameric complex, with four inward rectifier K^+^ channel subunits (Kir6.x) and four sulfonylurea receptor subunits (SUR), which together modulate cellular metabolism and membrane excitability. These channels are shown with two transmembrane domains and a central pore loop, flanked by significant cytoplasmic domains, essential for potassium ion selectivity. The SUR subunits exhibit 17 transmembrane domains and two nucleotide-binding folds, forming adenine nucleotide-binding sites (ABS1 and ABS2). In contrast, the mitochondrial KATP channel, formed by the interaction of CCDC51 with mitochondrial SUR (encoded by ABCB8), highlights a unique structure divergent from the plasma membrane counterpart. Cartoon in Figure 2 was created with BioRender.com.

The understanding of mitochondrial KATP channels has evolved over time, reflecting their complex nature and the ongoing research in this field. Initially, it was believed that these mitochondrial channels shared structural similarities with their plasma membrane counterparts, possibly being variants of the same family. Early studies suggested that subunits Kir6.1 or Kir6.2, which are characteristic of plasma membrane KATP channels, might also form the mitochondrial channels. However, this view has been challenged by more recent research. Subsequent investigations pointed towards the ROMK2 potassium channel, a variant of the renal outer medullary potassium channel, as a likely structural component of mitochondrial KATP channels [21,22]. This was a significant development, highlighting the distinct characteristics of mitochondrial channels. Furthermore, recent breakthroughs identified the CCDC51 gene as responsible for producing the pore-forming subunit of the mitochondrial KATP channel [23]. This finding was crucial in understanding the channel’s structure and function. The channel’s inhibition by glibenclamide, an antidiabetic drug, led to speculation about the involvement of the glibenclamide receptor (SUR) as a component of the channel. This hypothesis was confirmed by the discovery that CCDC51 interacts with mitochondrial SUR, encoded by the ABCB8 gene. This interaction forms a channel with the established pharmacological properties of mitochondrial KATP channels. Another intriguing aspect of mitochondrial KATP channels is the suggested involvement of ATP synthase subunits or respiratory chain components in their formation. This hypothesis gained support from observations that the channel is sensitive to specific modulators and inhibitors. It indicates that the F1FO segment of ATP synthase might facilitate potassium flux, functioning similarly for both potassium and hydrogen ions [24]. These findings underscore the possibility that mitochondrial ATP-sensitive potassium flux might be mediated by a variety of proteins, each contributing to the channel’s multifaceted role in cellular physiology. The structures of plasma membrane KATP channels and mitochondrial KATP channels are shown in Figure 2.

## 4. Mechanisms of Opening and Closing of KATP Channels

The regulation of plasma membrane KATP channels’ opening and closing is primarily governed by ATP and ADP levels but also influenced by various other physiological factors, including phosphatidylinositol 4,5-bisphosphate (PIP_2_), long-chain acyl-CoA molecules, and intracellular pH levels. This review delves into these diverse regulatory elements and explores how they collectively modulate KATP channel activation, particularly in the context of cancer cell physiology.

Plasma membrane KATP channels are inhibited by intracellular ATP, with binding occurring on the Kir6 subunit [25,26]. The inhibition is not due to phosphorylation but direct binding to the channel, with ATP analogues also inhibiting channel activity. In the absence of Mg^2+^, binding of ADP to SUR blocks KATP channel activity with lower affinity than ATP, highlighting the importance of electrostatic interactions with phosphate moieties of ATP [27,28]. Cancer cells undergo significant metabolic reprogramming, which includes a marked change in their ATP/ADP ratio. In normal cells, the adenine nucleotide translocator (ANT) facilitates the electrogenic exchange of ATP for ADP within mitochondria, a process closely linked to the mitochondrial membrane potential (ΔΨ). This exchange maintains a high cytosolic ATP/ADP ratio [29]. In contrast, cancer cells exhibit a significant decrease in this ratio owing to a switch to a different ATP/ADP exchange mechanism. Cancer cells predominantly utilize the ATP-Mg^2+^/phosphate carrier (AMPC) for ATP/ADP exchange, as opposed to ANT used in normal cells. AMPC differs from ANT in its ability to facilitate the net uptake of adenine nucleotides, essential for mitochondrial biogenesis, and is notably upregulated in cancer cells [30,31]. The non-electrogenic nature of exchange via AMPC leads to substantially lower ATP/ADP ratios in the cytosol of cancer cells, contributing to the characteristic aerobic glycolysis and lactate production, a phenomenon known as the Warburg metabolic phenotype [32]. The decreased ATP/ADP ratio in cancer cells has several repercussions, one of which is the potential activation of KATP channels. These channels are sensitive to intracellular ATP and ADP levels and play a critical role in various cellular processes. In the context of cancer, the reduced ATP/ADP ratio could lead to the activation of KATP channels, affecting cellular functions such as ion transport, membrane potential regulation, and possibly influencing cellular proliferation and survival mechanisms. Furthermore, the lower ATP/ADP ratios in cancer cells result in diminished stimulation of mitochondrial oxidative phosphorylation, favoring glycolysis over oxidative phosphorylation for ATP production. This shift in energy metabolism, however, does not seem to impede the growth and proliferation of cancer cells. The energy requirements for vital cellular processes like protein and nucleic acid synthesis in eukaryotic cancer cells are akin to those in prokaryotes, where natural ATP/ADP ratios are relatively low [33,34]. The alteration in ATP/ADP ratios in cancer cells, stemming from a switch in ATP/ADP exchange mechanisms, not only underscores the metabolic flexibility of cancer cells but also highlights potential targets for therapeutic intervention, such as the KATP channels. Understanding how these channels are regulated in the context of cancer metabolism and how they contribute to cancer cell physiology could open new avenues for cancer treatment strategies.

PIP_2_ is a crucial regulator of plasma membrane KATP channel activity, particularly influencing the Kir6.2 subunit. The binding of PIP_2_ to Kir6.2 facilitates the opening of KATP channels, a mechanism that appears to be conserved in Kir6.1 channels, underscoring the fundamental role of phospholipid binding and channel gating processes [35,36]. In the context of cancer cell signaling, PIP_2_ serves as a key substrate for generating second messengers, such as diacylglycerol (DAG) and inositol 1,4,5-trisphosphate (IP_3_), through the activation of phospholipase C (PLC). The dysregulation of PLC, leading to altered PIP_2_ metabolism, is a common feature in various oncogenic pathways [37]. This alteration in PIP_2_ levels is also intricately linked to the PI_3_K/AKT signaling pathway, often hyperactivated in cancers. The phosphorylation of phosphoinositides, including the conversion of PIP_2_ to its phosphorylated form PIP_3_, is modulated by oncogenic drivers like PI_3_K and tumor suppressors such as PTEN. PTEN acts as a phosphatase that converts PIP_3_ back to PIP_2_, with its loss or mutation leading to elevated PIP_3_ levels and subsequent aberrant activation of downstream signaling pathways that contribute to tumorigenesis [38]. Consequently, in cancer cells, where PIP_2_ levels are frequently upregulated, KATP channels are more likely to be in an activated state, suggesting a potential link between altered PIP_2_ metabolism and KATP channel activity in the cancerous milieu.

Long-chain acyl-coA (LC-CoA) molecules, which are intermediates in the β-oxidation of fatty acids, are known to activate plasma membrane KATP channels by interacting with the same residues on the Kir6.2 subunit as PIP_2_ [39,40]. In cancer cells, there is often a significant reprogramming of lipid metabolism, characterized by an increase in fatty acid synthesis and uptake to support rapid cell proliferation. This metabolic shift results in an accumulation of fatty acid intermediates like acyl-CoA molecules. Carracedo et al. have illustrated the pivotal role of lipid metabolism in cancer, emphasizing the synthesis and degradation of fatty acids, which subsequently influence the levels of acyl-CoA [41]. Consequently, the elevated presence of LC-CoA molecules in cancer cells is likely to contribute to the activation of KATP channels.

The activity of plasma membrane KATP channels is also influenced by the intracellular pH levels [42,43]. At a molecular level, the sensitivity of KATP channels to ATP is modulated by changes in pH. Studies involving the Kir6.2 subunit of these channels have identified critical amino acids, specifically Thr71 and His175, that are believed to be key in the channel’s pH sensitivity [44,45]. However, the precise mechanism by which these amino acids interact with protons and influence ATP-dependent channel gating remains an area of active research. Given the tendency of cancer cells to have a lower pH compared to normal cells, a consequence of their reliance on glycolysis for energy [46], there is a likelihood of increased KATP channel activity in these cells. The shift towards glycolysis in cancer cells, a process referred to as the Warburg effect, leads to increased production of lactic acid, thereby reducing the intracellular pH. This enhanced activity could play a role in the unique physiological behaviors observed in cancer cells, including their growth and survival mechanisms. Understanding this relationship between intracellular pH and KATP channel function could provide new insights into cancer cell biology and potential therapeutic targets.

Plasma membrane KATP channels are subject to regulation by phosphorylation. Specifically, Protein Kinase A (PKA) plays a key role in modulating the activity of these channels in smooth muscle and pancreatic tissues. The Kir6.2 subunit of the KATP channel contains two sites known to be targeted by PKA phosphorylation. When phosphorylated, these sites enhance the probability of the channel remaining open, altering its functional state [47,48]. Cancer cells often exhibit abnormal signaling via G protein-coupled receptors (GPCRs), leading to elevated cyclic AMP (cAMP) levels and consequent PKA activation. This aberration in GPCR signaling can stem from overexpression, mutations, or dysregulated control of these receptors, influencing adenylate cyclase (AC) activity and impacting downstream pathways [49].

In addition to PKA, Protein Kinase C (PKC) also regulates plasma membrane KATP channel activity. PKC achieves this through the phosphorylation of a conserved T180 residue on the Kir6.2 subunit [50,51]. In the context of cancer, PKC activation is often driven by increased diacylglycerol (DAG) and Ca^2+^ levels, common in dysregulated signal transduction pathways characteristic of malignancies. This increase in DAG may be a result of either heightened activity of phospholipase C (PLC) or changes in lipid metabolism typically observed in cancer cells [52]. The role of PKC in cancer is complex, with different isoforms playing either oncogenic or tumor-suppressive roles, depending on their expression and mutation status. Additionally, alterations in RTKs signaling, frequently observed in various cancers, can lead to PKC activation, thereby influencing cellular processes like proliferation, survival, and migration. 

**Figure 3:**
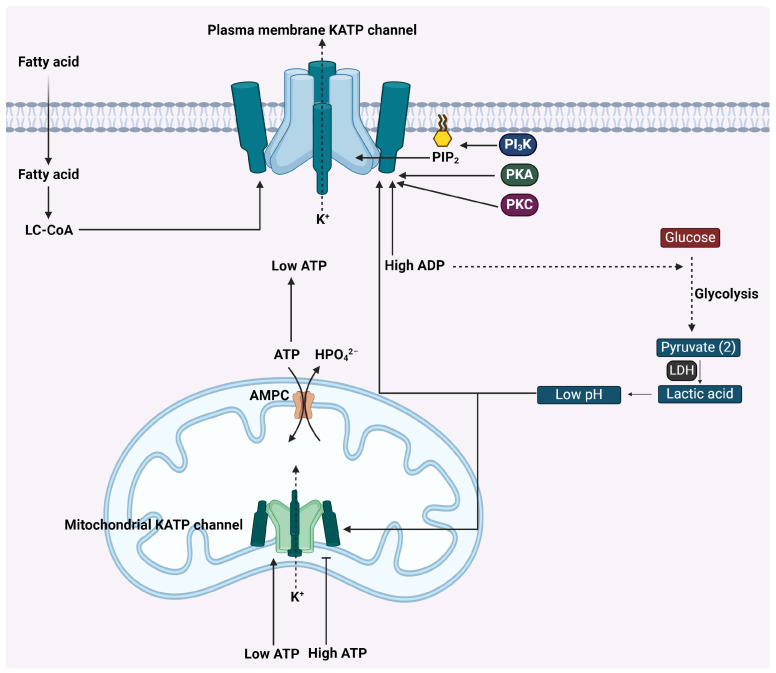
KATP Channel Regulation in Cell Metabolism. High concentration of ATP inhibits plasma membrane KATP channels at the Kir6 subunit, while ADP shows lower-affinity interaction at the SUR subunit, reflecting the distinctive ATP/ADP ratios in cells. PIP_2_ binding to Kir6.2 activates KATP channels, with further modulation by PKA and PKC phosphorylation amid cancerous signaling irregularities. Long-chain acyl-CoA molecules, elevated due to cancer cell lipid metabolism, also trigger KATP channel activation. Additionally, KATP channel activity is influenced by intracellular pH through specific amino acid interactions on Kir6.2, relevant in the acidic environment of cells from lactate production. Mitochondrial KATP channels are modulated by intracellular ATP levels, where a decrease prompts opening, and by pH changes, with acidity influencing their activity. Cartoon in Figure 3 was created with BioRender.com.

A notable distinction in the regulatory mechanisms of mitochondrial KATP channels, compared to their plasma membrane counterparts, is their activation in low-ATP conditions [23,53]. This is an adaptive response observed not only in cardiac tissues during ischemia but also in cancer cells, where a similar energy deficit can occur due to hypoxic conditions. The opening of mitochondrial KATP channels during energy scarcity helps to maintain cellular homeostasis. In the case of heart muscle cells, the onset of ischemia leads to a dip in ATP levels, which triggers the opening of these mitochondrial KATP channels. This event might be followed by a change in the mitochondrial membrane potential, which likely activates voltage-regulated potassium channels [54,55]. Furthermore, with ATP levels down, ion pumps may fail, causing an increase in cell calcium levels. The surge in calcium during ischemia and subsequent return of blood flow could overload the mitochondria with calcium, especially when oxygen is restored, potentially activating calcium-sensitive potassium channels [56]. This process is crucial as it facilitates the maintenance of ionic balance and cellular protection under stress. Unlike plasma membrane KATP channels, which are generally inhibited by a decrease in ATP, their mitochondrial equivalents are designed to respond to such energetic crises by opening, which underscores their role in cellular survival mechanisms. Moreover, the low-ATP condition induced by ischemia, a commonality shared with cancer cells experiencing hypoxia, underscores the universality of this protective response across different cell types. The metabolic stress in cancerous tissues can similarly activate mitochondrial KATP channels, suggesting a potential target for therapeutic intervention. Aside from ATP concentration, mitochondrial KATP channels are influenced by a host of other factors. These include the mitochondrial membrane potential, intracellular pH, and the presence of reactive oxygen species (ROS) [53,57]. Each of these factors can modulate the activity of mitochondrial KATP channels, contributing to their complex role in cell physiology. For instance, a change in the mitochondrial membrane potential could activate or deactivate these channels, while a more acidic intracellular pH, a result of anaerobic glycolysis and ATP hydrolysis, may modify their sensitivity. Additionally, an increase in ROS during reperfusion, when oxygen is reintroduced, has been noted to stimulate these channels, suggesting a multifaceted regulatory system that responds to a variety of cellular conditions. Figure 3 presents a model of the regulatory mechanisms governing the opening and closing of KATP channels.

## 5. Changes in Expression or Mutation of Genes Constituting KATP Channels in Cancer Cells

Understanding the expression and mutations of KATP channel genes in cancer cells is vital for assessing disease progression, developing targeted therapies, enabling personalized medicine, understanding drug resistance mechanisms, identifying diagnostic and prognostic biomarkers, and gaining insights into cancer metabolism.

Research has demonstrated varying expression patterns of the ABCC8 and ABCC9 genes, crucial in forming KATP channels, across different cancer types. Notably, ABCC8 gene expression exhibits both downregulation and upregulation in cancers such as pancreatic, breast, lung, and colorectal. This review will further explore the varied expression changes in ABCC8 and ABCC9 in a range of cancers, highlighting their potential impact on cancer behavior and treatment responses.

Mohelnikova-Duchonova and colleagues, in their study employing quantitative real-time PCR (qPCR) on tissue samples from 32 patients undergoing surgery for pancreatic adenocarcinoma (PDAC), observed downregulation in the expression of the ABCC8 gene [58]. Hlaváč et al. conducted transcript level analysis of 49 human ABC transporters and immunoblotting for protein expression on post-treatment tumor and non-neoplastic tissue samples from 68 breast carcinoma patients, along with an independent series of 100 pretreatment patients, revealing downregulation of ABCC8 in breast carcinoma [59]. Wang et al. analyzed the FPKM dataset from the TCGA-LUAD database, using 535 lung adenocarcinoma (LUAD) samples and 59 paracancerous samples, and found downregulation of ABCC8 in lung adenocarcinoma [60]. Hlavata et al. conducted quantitative real-time polymerase chain reaction (qRT-PCR) on tissue samples from colorectal carcinoma (CRC) patients, revealing downregulation of ABCC8 in colorectal carcinoma [61]. Conversely, Huang et al. utilized immunocytochemistry and Western blot techniques on human glioma cell lines (U87 and U251), glioma biopsies, and a mice tumor model and observed upregulation of ABCC8 in glioma, impacting cell proliferation and ERK activity [62]. Mao X. et al. analyzed the mRNA expression of ABCC family members in 882 gastric cancer (GC) patients and found upregulation of ABCC8 in gastric cancer [63]. Xiao et al. conducted gene expression analysis using databases on Pancreatic Endocrine Neoplasms (PanNETs) tissues and found upregulation in ABCC8 in PanNETs [64]. 

**Figure 4:**
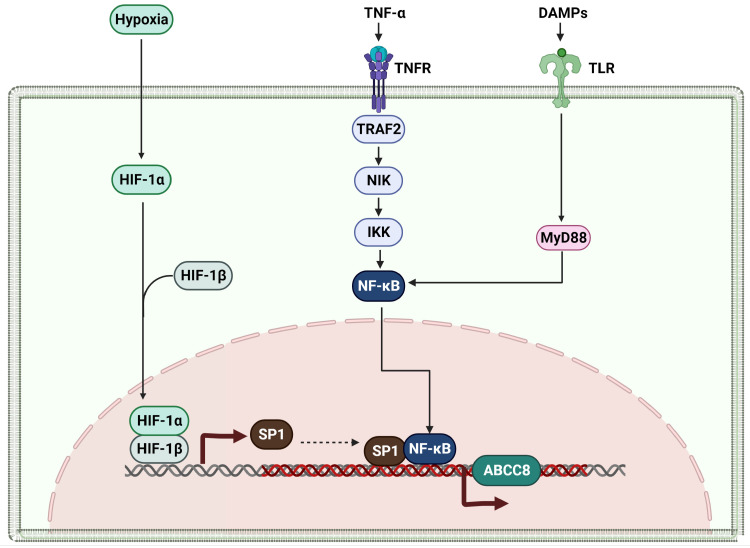
Regulation of ABCC8 Expression in Hypoxic and Inflammatory Conditions. Hypoxia-induced stabilization of HIF-1α interacts with HIF-1β, initiating ABCC8 transcription via SP1 activation. In parallel, TNF-α engagement with TNFR triggers the NF-κB signaling pathway, enhancing ABCC8 expression. Additionally, TLR4 activation by DAMPs, leading to MyD88 involvement, further influences ABCC8 transcription. These interconnected pathways underscore the complexity of ABCC8 regulation in the cancer microenvironment, driven by hypoxia and inflammation commonly associated with tumor progression. Cartoon in Figure 4 was created with BioRender.com.

Research focusing on the modulation of ABCC8 expression in cancer cells is currently insufficient. Earlier studies have concentrated on understanding the regulatory mechanisms of ABCC8 expression, specifically in pancreatic β-cells within diabetic models [65,66]. Key transcription factors such as Sp1, FoxA2/HNF3β, Beta2/NeuroD, and STAT3 have been recognized as vital regulators of ABCC8 transcription in these cells [67,68]. In the realm of the central nervous system (CNS), studies have shown that hypoxia or ischemia instigates an increase in ABCC8 expression, a process closely related to SP1 activation by hypoxia-inducible factor 1α (Hif1α) [69]. Sp1 is known to initiate ABCC8 transcription across various species [66,69], and targeted inhibition or genetic downregulation of Hif has been found to mitigate brain ischemia/hypoxia by reducing SUR1 overexpression [70,71]. In cancer cells, hypoxia often occurs due to rapid tumor growth surpassing blood vessel development, leading to a reduction in oxygen supply. This decrease in oxygen is likely to stabilize Hif1α, which in turn is expected to activate SP1 and subsequently induce ABCC8 in response to the hypoxic state. Additionally, the activation of ABCC8 transcription in the CNS via the NF-κB pathway, triggered by TNFα, plays a significant role, with NF-κB binding sites present in the ABCC8 promoter regions of both rats and humans [72]. In brain endothelial cells, TNFα exposure, acting as an NF-κB stimulator, enhances ABCC8 mRNA and SUR1 protein levels [73]. Moreover, TLR4 pathway activation in microglia leads to an increase in ABCC8 mRNA and protein levels in the CNS [74,75]. Based on insights from studies in pancreatic beta cells and the CNS, it is hypothesized that ABCC8 expression in cancer cells may be significantly influenced by mechanisms involving hypoxia and inflammation, mediated by TNF-α and TLR signaling. Concurrently, cancer-related inflammation, often a result of interactions between tumor cells and the immune system, prompts the secretion of various factors that stimulate immune cells to produce TNF-α. This cytokine is speculated to activate the NF-κB pathway, playing a pivotal role in inflammatory responses and the regulation of ABCC8 expression in cancer cells. Furthermore, TLR signaling within cancer tissues, often initiated by the recognition of tumor-associated antigens or damage-associated molecular patterns (DAMPs) released from stressed or dying cancer cells, is expected to be an additional factor influencing ABCC8 expression. The mechanism that controls the expression of the ABCC8 gene in cancer cells is illustrated in Figure 4.

Next, this review will examine the findings from research on mutations in the ABCC8 gene. Xiao et al. undertook an extensive mutation analysis using databases focused on PanNETs tissues. This investigation led to the discovery of a notable mutation in the ABCC8 gene specific to PanNETs [64]. In a case study presented by Calton et al., genetic analysis of a child diagnosed with hepatoblastoma revealed a recessive mutation in the ABCC8 gene [76]. Additionally, this study identified UPD 11p15, a genetic abnormality, in both the pancreas and liver of the patient. Further advancing understanding, Soucek et al. [77]. conducted comprehensive exome sequencing in breast cancer patients. Their approach, which involved next-generation sequencing, specifically targeted the ABCC8 and ABCD2 genes. Remarkably, they uncovered 113 genetic mutations within these genes, including a range of frameshifts and missense alterations, shedding new light on the genetic landscape of breast cancer.

Regarding investigations into the expression of the ABCC9 gene, Vázquez-Sánchez et al. conducted research on cervical cancer, analyzing both cell lines and human biopsy samples. Their methodology involved reverse transcription polymerase chain reaction and immunochemistry. The findings highlighted upregulation of ABCC9 in cervical cancer [4]. Mao et al. undertook an extensive study with 882 gastric cancer (GC) patients. Their focus was on the mRNA expression of the ABCC family, including ABCC9. The results indicated upregulation of ABCC9 in gastric cancer [63]. In another study by Mao et al., the emphasis was on epithelial ovarian cancer (EOC). They employed quantitative real-time PCR to analyze ABC and SLC transporter genes in EOC tissues, revealing upregulation of ABCC9 in epithelial ovarian cancer [78]. However, contrary to the above results indicating upregulation of ABCC9 expression in various cancers, there have also been reports of studies showing its downregulation. For instance, Zhang et al. reported downregulation of ABCC9 in triple-negative breast cancer based on differential expression and methylation analysis of the tissue [79]. Similarly, Demidenko et al. found downregulation of ABCC9 in prostate cancer, as determined through gene expression profiling and methylation analysis of prostate cancer tissue [80]. Additionally, mutations in ABCC9 have been documented in diverse cancers such as large granular lymphocyte leukemia, endometrial, and gastric cancers (Cheon et al. [81], Le Gallo et al. [82], Zhang et al. [83]), expanding our understanding of its genetic variability in oncogenesis.

In another notable study, Warnecke-Eberz et al. identified upregulation of the KCNJ8 gene in biopsies from patients with locally advanced esophageal squamous cell carcinoma (ESCC), employing genome microarray and TaqMan low-density array techniques for their analysis [84]. Furthermore, research conducted by Zhang et al. revealed upregulation of the KCNJ11 gene via NF-κB signaling in hepatocellular carcinoma (HCC), a finding observed across multiple HCC cell lines, including Hep3B, MHCC-97H, MHCC-97L, Huh7, SUN-423, and HepG2. This study utilized data mining of the TCGA cohort to arrive at its conclusions [85]. 

In our final topic, we delve into the gene expression of CCDC51, a key component of mitochondrial KATP channels. The body of research specifically focusing on the CCDC51 gene is quite scarce. However, a significant piece of research employing analysis of both RNA and protein levels has provided insight into the CCDC51 gene, which encodes a subunit integral to the mitochondrial KATP channels. This study utilized existing datasets to ascertain that CCDC51 is actively transcribed and translated across a wide array of tissues in both humans and mice, indicating a fundamental role in cellular physiology [86,87]. Further detailed examination through immunofluorescence assays has shed light on the spatial expression pattern of the CCDC51 protein within cells. These assays revealed a precise localization, with the protein consistently present on the inner mitochondrial membrane [23,88]. This finding is notable as it underscores the specificity of CCDC51’s role in mitochondrial function. Moreover, the same assays have verified that the outer mitochondrial membrane lacks CCDC51 protein presence, which suggests selective involvement in the inner mitochondrial mechanisms. This selective localization is particularly evident in HeLa cells, a line of human cervical cancer cells, thereby providing a window into the gene’s functional dynamics in a cancerous context. The information discussed thus far is concisely summarized in Table 1 for ease of reference.

## 6. Cancer Cell Growth and KATP Channel

The regulation of membrane potential, essential for cell cycle progression, involves adaptations in membrane permeability. K^+^ conductance is a key factor in setting the resting membrane potential across various cell types. Unlike the quick action potentials in neurons, cell cycle-related changes in potential are more gradual, a result of alterations in K^+^ conductance [89,90]. In lymphocytes and Schwann cells, research indicates that blocking K^+^ channels can lead to a halt in the cell cycle or reduce proliferation [91,92]. Studies on embryonic retinal cells have shown alterations in K^+^ channel composition during the G1 phase [93]. Furthermore, in mouse oocytes, K^+^ channel activity appears to be partly governed by the cytoplasmic cell-cycle clock, suggesting a role in cell division beyond nuclear controls [94]. This implies K^+^ channels’ involvement in cell-cycle checkpoint signaling and the integration of cellular clocks. K^+^ channels also influence the entry of Ca^2+^ into cells, pivotal for cell proliferation and other key physiological processes [95,96]. The current understanding extends beyond K^+^ currents alone as emerging evidence points to specific channels impacting cell proliferation through non-standard mechanisms like protein interactions and voltage-induced conformations.

This review focuses on the role of KATP channels in cell cycle progression and cancer cell proliferation. KATP channels, linking cellular metabolism to membrane excitability, are gaining attention for their involvement in tumor growth and cancer cell proliferation. However, this role is still being unraveled. For instance, Scarth et al. observed a crucial role for KATP channels in cervical carcinogenesis related to human papillomaviruses (HPVs), with a noted upregulation in the SUR1 component in HPV-positive cervical cancer cells, correlated with E7 oncoprotein activity. Blocking these channels significantly reduced cell proliferation, suggesting a new avenue for HPV-related cervical cancer treatment [97]. Ru et al. found that KATP channel blockers reduced proliferation and tumor growth in U87-MG human glioma cells, hinting at a Ca^2+^-dependent mechanism [98]. Huang et al. also noted higher KATP channel expression in glioma tissues compared to normal ones. Their inhibition lessened glioma cell proliferation and tumor formation in animal models, indicating the potential of KATP channel blockers in glioma therapy [62]. Klimatcheva et al. identified a KATP channel in MCF-7 human breast cancer cells, essential for G1 phase progression, presenting new targets for breast cancer treatment [99]. Wondergem et al. discovered that the sulfonylurea receptor and KATP channels regulate cell growth in human bladder carcinoma (HTB-9) cells. Glibenclamide, a KATP channel blocker, effectively reduced cell proliferation, underscoring the role of the channel role in cancer cell growth mechanisms [100]. Wonderlin et al. reported that membrane potential changes in MCF-7 cells during the cell cycle, likely related to K^+^ permeability, are linked to cell cycle progression in breast cancer cells [101]. These studies collectively emphasize the significant role of KATP channels in various cancers, particularly in cell cycle progression and proliferation. The specific mechanisms, including their impact on intracellular Ca^2+^ signaling and potential activation of the MAPK/ERK pathway, remain areas of active research. This growing body of evidence opens up new possibilities for cancer therapy targeting KATP channels. Further comprehensive research is needed to fully understand and exploit KATP channels as therapeutic targets in cancer.

Exploring the function of mitochondrial KATP channels, Angela Paggio’s research offers crucial insights, particularly in cancer cell biology. Paggio’s team employed Crispr/Cas9 to specifically target the CCDC51 gene in HeLa cells, which is vital for these channels. This gene deletion led to a disruption of ATP-dependent potassium movements within the mitochondria, causing continuous swelling, a process usually regulated by mitochondrial KATP channels [23]. This study delves into KATP, with a focus on their presence and function in mitochondria. It introduces MITOK, a novel protein complex, and details its role in ATP-sensitive K^+^ transport within the mitochondrial KATP channel. The paper clarifies how MITOK and MITOSUR proteins control the mitochondrial KATP channel, emphasizing its importance in cardioprotection, cellular death regulation, and maintaining metabolic balance. The research is notable for its comprehensive molecular analysis of the mitochondrial KATP channel’s functionality in the mitochondrial membrane, significantly advancing our understanding of mitochondrial physiology and related diseases. The CCDC51-depleted cells displayed unique ring-shaped mitochondria and underwent spontaneous transient depolarizations. This was accompanied by a noticeable decrease in oxygen consumption rates, pointing to reduced mitochondrial efficiency. Electron microscopy studies showed that these cells had enlarged cristae, indicative of structural changes due to altered matrix volume and osmotic balance. Furthermore, the CCDC51-lacking cells exhibited heightened ROS production, signaling compromised mitochondrial function and a shifted redox state. In cancer cells like HeLa, such mitochondrial swelling and dysfunction can profoundly affect metabolism, which relies heavily on mitochondria for both energy production and controlling cell death. The resulting oxidative stress from altered redox balance and increased ROS could interfere with cellular signaling and DNA stability, factors critical in cancer development and resistance to treatment. The study’s findings that CCDC51 is essential for mitochondrial response to cellular stress have important implications in cancer cell biology. By influencing redox balance and maintaining mitochondrial structure, CCDC51 plays a key role in managing how cancer cells survive and adapt to stress, highlighting its potential as a therapeutic target in cancer treatment.

## 7. Exploring the Role of KATP Channel Activators in Cancer Biology

In cancer biology, activators and inhibitors of KATP channels are crucial as they can regulate cancer cell survival, metabolism, and response to therapies by modulating mitochondrial functions and cellular ion balance. Table 2 outlines a range of substances that either activate or inhibit KATP channels located in the plasma membrane and mitochondria. Certain compounds exert their effects on both types of KATP channels, whereas others are selective in their action, targeting either plasma membrane or mitochondrial channels exclusively.

The function of KATP channel activators is to stimulate the opening of these channels. When KATP channels open, K^+^ ions flow out of the cell, leading to hyperpolarization of the cell membrane. This hyperpolarization can affect many cellular activities, including the regulation of insulin secretion in pancreatic cells, dilation of blood vessels, and protection of cardiac tissue during periods of metabolic stress. Types of KATP channel activators include diazoxide, minoxidil, Pinacidil, and Cromakalim. The exploration of KATP channel activators in cancer research has unfolded a complex landscape where drugs interact diversely with various cancer cell types. Each drug’s unique impact offers a glimpse into the intricate relationship between ion channels and cancer cell behavior.

In a study by Wondergem et al., diazoxide was shown to stimulate growth in human bladder carcinoma cells, as evidenced by increased protein accumulation [100]. However, this stimulation did not translate to an increase in cell number. The research implies that diazoxide’s role in cancer cell growth might be more nuanced, possibly affecting certain cellular processes without necessarily promoting cell proliferation.

Several studies have shed light on the diverse impacts of minoxidil on cancer cells. Maqoud et al. found that, in renal tumors and canine breast cancer, minoxidil led to elevated expression of the Sur2A subunit in proliferating cells, suggesting a possible role in cancer progression [102]. Meanwhile, Qiu et al. demonstrated that minoxidil could reduce invasion in human breast cancer cells in a dose-dependent manner, with its effects being amplified when used in combination with ranolazine [103]. In studies by Gu et al., minoxidil was shown to increase blood–tumor barrier (BTB) permeability in a rat brain tumor model, indicating its potential in enhancing drug delivery to tumors. This effect was further elaborated upon by Ningaraj et al., who suggested that minoxidil could improve the delivery of other anti-neoplastic agents to brain tumors, potentially enhancing their efficacy [104]. Kim et al.’s study uniquely positions Pinacidil as an apoptosis inducer in HepG2 human hepatoblastoma cells, highlighting its potential for selectively targeting cancer cells [105]. Lee et al.’s research presents Cromakalim as an anti-tumor agent, inhibiting the growth of human neuroblastoma and astrocytoma cell lines [106]. This effect, counteracted by sulfonylureas, underscores Cromakalim’s specific action on the KATP channel. Malhi et al. found that Cromakalim stimulated DNA synthesis in liver cells, suggesting a role in liver regeneration and growth control, with significant implications for liver cancer therapy and liver regeneration research [107]. Table 3 presents the biological impacts of KATP channel activators on cancer cells.

In conclusion, these studies collectively highlight the significant roles of KATP channel activators in cancer biology. They underscore the potential of these drugs in managing various aspects of cancer progression, from cell proliferation to metastasis control and drug delivery enhancement. The insights gained are crucial for developing novel therapeutic strategies, yet the complexity of their mechanisms and varied responses across cancer types necessitate more detailed investigations. As research delves deeper, the prospects for targeted cancer therapies appear increasingly promising.

## 8. Exploring the Role of KATP Channel Inhibitors in Cancer Biology

The intricate relationship between KATP channel inhibitors and cancer cell dynamics has been the subject of extensive research, revealing the multifaceted roles these compounds play in oncology. Among these, sulfonylurea (SU) inhibitors, originally developed for diabetes management, have shown surprising efficacy against various cancer types.

Glyburide, a prominent SU inhibitor, has demonstrated notable anti-cancer properties. In a study by Wondergem et al., it was found to reduce cell proliferation in human bladder carcinoma cells (HTB-9) by altering cell cycle distribution, particularly decreasing the proportion of cells in the S phase and increasing those in the G0/G1 phase [100]. This indicates that Glyburide has the potential to regulate cell growth through KATP channels, influencing the central mechanisms of the cell cycle. In another significant study by Li et al., Glyburide significantly reduced lung tumor incidence and severity in a mouse model, potentially through its inhibition of the NLRP3 inflammasome, a key player in inflammation and cancer development [108]. This dual action against both cell proliferation and inflammation underscores Glyburide’s potential as a versatile anti-cancer agent. Sun et al. demonstrated that Glyburide at concentrations of 100–1000 μM in PC3 human prostate cancer cells induces PLC-dependent Ca^2+^ rises involving ER release and also triggers Ca^2+^-independent cell death [109].

Glipizide, another SU-class drug, has shown efficacy in breast cancer treatment, especially when used in combination with Atrial Natriuretic Peptide (ANP). Mao et al. discovered that this combination more effectively inhibited breast cancer growth and metastasis in MMTV-PyMT mice than Glipizide alone [110]. Their research also highlighted Glipizide’s ability to impede tumor-induced angiogenesis, a critical factor in cancer progression, by inhibiting VEGF/VEGFR2 signaling in human umbilical vein endothelial cells. Additionally, in the study by Nazim et al., Glipizide was found to enhance TRAIL-mediated apoptotic cell death in human lung adenocarcinoma cells. This was achieved by downregulating p-Akt and p-mTOR and promoting autophagy flux activation, indicating its potential to overcome TRAIL resistance in cancer cells [111]. In addition, studies by Cuiling Qi et al. have shown that Glipizide acts as a potent inhibitor of tumor angiogenesis [112]. In prostate cancer, studies by Cuiling Qi et al. revealed that Glipizide significantly reduces microvessel density in tumor tissues, highlighting its potential in anti-angiogenic therapy [113]. Long et al. synthesized glimepiride–metformin adduct (GMA) and showed that it not only inhibited the viability of breast cancer cells more effectively than either glimepiride or metformin alone but also induced G_1_/S phase cell cycle arrest and apoptosis. This was achieved through the activation of AMPK and the modulation of p53, p21, cyclin D1, and CDK4 expression, highlighting GMA’s potential as a therapeutic option for breast cancer in diabetic patients [114].

In addition to these compounds, Shuai Li et al. demonstrated that Gliclazide reduces colitis-associated colorectal cancer formation by decreasing colonic inflammation and regulating the AMPK-NF-κB signaling pathway, suggesting its potential as a preventive treatment for colitis-associated colorectal cancer [115].

The nitrated form of Nateglinide, NO_2_-NAT, as studied by Koji Nishi et al., has shown promising results as a novel nitric oxide-based chemotherapeutic agent. It induces apoptosis in human pancreatic cancer cells through the release of nitrate and nitrite ions, thereby increasing extracellular lactate dehydrogenase leakage and annexin-positive cells [116]. This study adds to the growing body of evidence supporting the repurposing of diabetic drugs for cancer treatment, leveraging their unique mechanisms of action to target various aspects of cancer cell physiology. 

**Figure 5:**
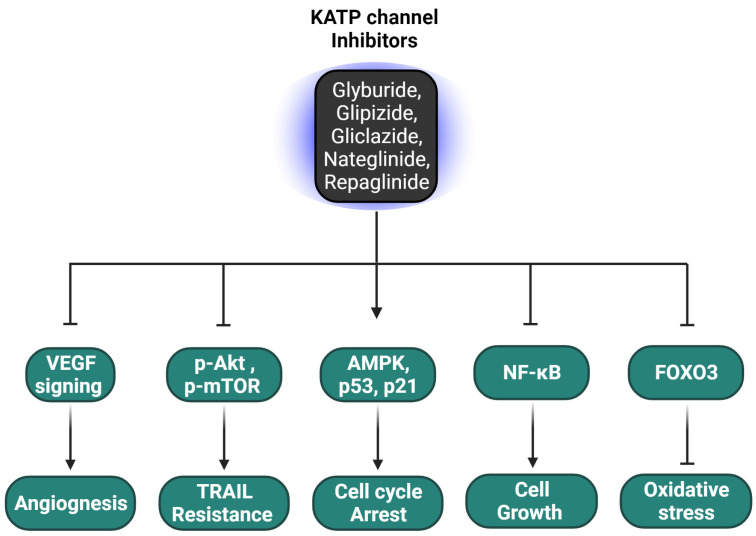
KATP Channel Inhibitors and Their Effects on Cancer Cell Signaling. KATP channel inhibitors, including Glyburide, Glipizide, Gliclazide, Nateglinide, and Repaglinide, modulate key signaling pathways in cancer cells. These agents impact angiogenesis through VEGF signaling, induce TRAIL resistance via the p-Akt and p-mTOR pathways, promote cell cycle arrest by activating AMPK and enhancing p53/p21, inhibit cell growth through NF-κB pathways, and affect oxidative stress management through the FOXO3 axis. The representation underscores the multifaceted roles of KATP channel inhibitors as modulators of cancer cell dynamics. Cartoon in Figure 5 was created with BioRender.com.

Lastly, Repaglinide, a drug identified from a drug library screen, emerged as an effective FOXO3 inhibitor in neuroblastoma treatment. Salcher et al. demonstrated that Repaglinide effectively suppresses FOXO3-mediated cellular migration by inhibiting FOXO3’s binding to the LUM promoter, thereby reducing lumican RNA and protein expression [117]. This finding suggests Repaglinide’s potential role in targeting aggressive tumor behaviors linked to FOXO3. Figure 5 and Table 4 present the biological impacts of KATP channel inhibitors on cancer cells.

Overall, the research in this field encourages a broader perspective on existing drugs and their potential applications, advocating for continued exploration and clinical trials to fully harness their capabilities in cancer treatment. The repurposing of these drugs not only accelerates the availability of new cancer treatments but also exemplifies the efficiency and cost-effectiveness of leveraging existing pharmacological knowledge for novel therapeutic purposes.

## 9. Conclusions

In summary, the exploration of KATP channels as potential therapeutic targets in cancer treatments has revealed new horizons in oncology. The unique regulatory functions of these channels in cancer cell metabolism and electrical signaling offer innovative avenues for intervention. However, the journey from theoretical understanding to practical application in clinical settings is fraught with challenges and opportunities.

The promise of KATP channels in cancer therapy lies in their ability to modulate crucial cellular processes, which are often dysregulated in cancer cells. By targeting these channels, there is the potential to disrupt cancer cell proliferation and survival selectively. However, this approach requires a nuanced understanding of the diverse roles that KATP channels play in different types of cancer as their function can vary significantly depending on the tumor environment and cancer cell type [97].

One of the major challenges in targeting KATP channels is the risk of adverse effects on normal cells given the channels’ presence and importance in various tissues. This necessitates the development of strategies that can selectively target cancer cells while minimizing harm to normal cells. Additionally, the variability in response to KATP channel-targeted therapies among patients highlights the need for personalized medicine approaches in cancer treatment.

Investigations into ATP-sensitive potassium channels within plasma membranes have greatly enriched our knowledge regarding cell function and drug action. However, exploring mitochondrial KATP channels is becoming an essential aspect of cancer research. These channels are key to regulating mitochondrial operations, influencing essential functions like ATP generation, programmed cell death, and defense mechanisms against physiological stress. Strategically influencing these channels presents a viable approach for selectively targeting cancer cells for destruction while preserving normal cells, underscoring the importance of dedicated research in this domain. Progressing research on mitochondrial KATP channels poses distinct challenges, including understanding the complex nature of mitochondrial biology, achieving targeted modulation, and crafting precise agents that specifically affect mitochondrial channels without impacting those in the plasma membrane. Furthermore, transporting drugs into mitochondria is challenging due to their protective double membrane and the requirement to traverse the cellular interior. Overcoming these hurdles necessitates a focused endeavor to clarify the characteristics of mitochondrial KATP channels, innovate drug transport mechanisms, and synthesize targeted modulators. Such progress will not only deepen our grasp of how mitochondria function in cancer but also pave the way for developing treatments that are both more effective and safer. 

## Figures and Tables

**Table 1:** Variations in Expression and Mutations of KATP Channel Genes (ABCC8, ABCC9, KCNJ8, and KCNJ11) Across Different Cancer Types.

Cancer Cell Type	Model	Research Method	Expression or Mutation	Ref.
Pancreatic Adenocarcinoma (PDAC)	Patient tissue samples (32 surgically treated PDAC patients)	Quantitative Real-Time PCR (qPCR)	Downregulation of ABCC8 in PDAC tumors	[58]
Breast Carcinoma	Post-treatment tumor and non-neoplastic tissue samples from 68 patients; independent series of 100 pretreatment patients	Transcript level analysis of 49 human ABC transporters; immunoblotting for protein expression	Downregulation of ABCC8 in Breast Carcinoma	[59]
Lung Adenocarcinoma (LUAD)	Patient samples (535 LUAD samples and 59 paracancerous samples from TCGA database)	Analysis using FPKM dataset from TCGA-LUAD	Downregulation of ABCC8 in LUAD cancer	[60]
Colorectal Carcinoma (CRC)	Tissue samples from CRC patients	Quantitative real-time polymerase chain reaction (qRTPCR)	Downregulation of ABCC8 in CRC	[61]
Glioma	Human glioma cell lines (U87 and U251), glioma biopsies, and a mice tumor model	Immunocytochemistry, Western blot	Upregulation of ABCC8 in Glioma	[62]
Gastric Cancer (GC)	882 GC patients	Analysis of mRNA expression of ABCC family members in GC patients	Upregulation of ABCC8 in GC	[63]
Pancreatic Endocrine Neoplasms (PanNETs)	PanNET tissues	Gene expression analysis and mutation identification using databases	Upregulation and Mutation in ABCC8 in PanNETs	[64]
Hepatoblastoma	Case study of a child with hepatoblastoma	Genetic testing for ABCC8 mutation and UPD 11p15	ABCC8 recessive mutation and UPD 11p15 in pancreas and liver	[76]
Breast Cancer	Exome sequencing in breast cancer patients	Next-generation sequencing of ABCC8 and ABCD2 genes	113 genetic mutation in ABCC8 and ABCD2, including frameshifts and missense alterations	[77]
Cervical Cancer	Cervical cancer cell lines and human biopsies	Reverse transcription polymerase chain reaction and immunochemistry	Upregulation of ABCC9 in Cervical Cancer	[4]
Gastric Cancer (GC)	882 GC patients	Analysis of mRNA expression of ABCC family members in GC patients	Upregulation of ABCC9 in GC	[63]
Epithelial Ovarian Cancer (EOC)	EOC tissue	Quantitative real-time PCR of ABC and SLC transporter genes in EOC	Upregulation of ABCC9 in EOC	[78]
Triple-negative Breast Cancer	Triple-negative Breast Cancer tissue	Differential expression and methylation analysis	Downregulation of ABCC9 in Breast Cancer	[79]
Prostate Cancer	Prostate Cancer tissue	Gene expression profiling and methylation analysis	Downregulation of ABCC9 in Prostate Cancer	[80]
Large Granular Lymphocyte (LGL) Leukemia	105 (LGL) Leukemia patients	Whole-exome and transcriptome sequencing	ABCC9 Mutation: Identified as a recurrently mutated putative driver	[81]
Endometrial Cancer	Frozen primary tumor tissues	Whole-exome sequencing	ABCC9 Mutation: 6% of serous tumors	[82]
Gastric Cancer (GC)	Genomic variant analysis with TCGA database	Prognosis model construction based on TCGA gastric cancer data	ABCC9 Mutation	[83]
Esophageal cancer	Patients with locally advanced squamous cell carcinoma of the esophagus (ESCC) biopsies	Genome microarray and TaqMan low-density array	Upregulation of KCNJ8	[84]
Hepatocellular carcinoma (HCC)	HCC cell lines Hep3B, MHCC-97H, MHCC-97L, Huh7, SUN-423, and HepG2	Data mining TCGA cohort	Upregulation of KCNJ11 via NF-κB signaling	[85]
Human cervical cancer	Cell lines HeLa	Immunofluorescence assay	Expression of CCDC51 on mitochondrial inner membrane	[23]

**Table 2:** Regulators of KATP channels and their specificity for mitochondrial versus plasma membrane channels.

Action on KATP Channels	Plasma Membrane KATP Channel	Plasma Membrane and Mitochondrial KATP Channel	Mitochondrial KATP Channel
Activator	P-1075 MCC-134	Cromakalim Pinacidil P-1060 Sildenafil Isoflurane Aprikalim Minoxidil Sulfate	Diazoxide Nicorandil BMS 191095
Inhibitor	HMR1098 (1833) Glimepiridec	Glibenclamide Glipizide	5-Hydroxydecanoate MCC-134

**Table 3:** Impact of KATP Channel Activators on Cancer Cell Dynamics and Tissue Responses.

Action on KATP Channels	Drug	Treatment Concentration	Model	Results	Ref.
Activator	Diazoxide	10 μM	Human Bladder Carcinoma (HTB-9)	Stimulated growth measured by protein accumulation but did not increase cell number.	[100]
Activator	Minoxidil	0.777–77.7 mg/kg/day	Renal tumor in male rats and Breast cancer in female dogs	Elevated immunohistochemical reactivity to Sur2A-mAb in cytosol of Ki67^+^/G3 cells in renal tumor. Elevated expression of Sur2A subunit in proliferating cells in breast cancer.	[102]
Activator	Minoxidil	2.5, 5 and 50 μM	MDA-MB-231, MDA-MB-468 (Triple-negative human breast cancer)	No effect on cell viability and proliferation. Reduced invasion in a dose-dependent manner.	[103]
Activator	Minoxidil + Ranolazine	0.625 μM Ranolazine + 2.5 μM minoxidil	MDA-MB-231, MDA-MB-468	Significant additive anti-invasive effects at low concentrations.	[103]
Activator	Minoxidil Sulfate (MS)	30 μg/kg/min for 15, 30, and 60 min	Rat brain glioma (C6)	Increased expression of caveolin-1 protein at tumor sites. Peak expression at 15 min of MS perfusion. Increased BTB permeability potentially mediated by ROS.	[104]
Activator	Pinacidil	1 mM	HepG2 human hepatoblastoma cells	Increase apoptosis.	[105]
Activator	Cromakalim	200 μM for SK-N-MC and 600 μM for U-373 MG	SK-N-MC human neuroblastoma, U-373 MG human astrocytoma cells	Inhibition of intracellular Ca^2+^ signaling. Inhibited growth of SK-N-MC and U-373 MG cell lines.	[106]
Activator	Cromakalim	5 μM	Primary rat hepatocytes, Human cancer cell lines	Increased cellular DNA synthesis in rat hepatocytes and human liver cell lines.	[107]

**Table 4:** Impact of KATP Channel Inhibitors on Cancer Cell Dynamics and Tissue Responses.

Action on KATP Channels	Drug	Treatment Concentration	Model	Results	Ref.
Inhibitor	Glyburide	75 and 150 μM for 48 hr	Human Bladder Carcinoma (HTB-9)	Reduced cell proliferation. Increased percentage of cells in G_0_/G_1_ phase; reduced percentage in S phase.	[100]
Inhibitor	Glyburide	10 μL/g body weight	B(a)p + LPS-induced mouse lung cancer	Reduced lung tumor incidence. Lower expression of NLRP3, IL-1β, and Cleaved-IL-1β proteins.	[108]
Inhibitor	Glyburide	100–1000 μM	PC3 human prostate cancer cells	Induced Ca^2+^ rises were PLC-dependent and involved Ca^2+^ release from the ER. Also caused Ca^2+^-independent cell death.	[109]
Inhibitor	Glipizide	5 mg/kg	MMTV-PyMT mice, HUVECs	Inhibited breast cancer growth and metastasis in MMTV-PyMT mice by suppressing VEGF/VEGFR2 signaling.	[110]
Inhibitor	Glipizide	0, 25, 50, and 100 μM	Human lung adenocarcinoma cells	Triggered TRAIL-mediated apoptotic cell death. Downregulated p-Akt and p-mTOR.	[111]
Inhibitor	Glipizide	2, 4, and 8 μg	Chick embryo CAM and YSM models, Xenograft tumor and MMTV-PyMT transgenic mouse models	Significantly inhibited blood vessel formation and development. Suppressed tumor angiogenesis, growth, and metastasis.	[112]
Inhibitor	Glipizide	5 mg/kg	TRAMP transgenic mouse model, Human umbilical vein endothelial cells	Suppressed prostate cancer growth and metastasis. Significantly reduced microvessel density in tumor tissues without inhibiting cell proliferation.	[113]
Inhibitor	Glimepiride	0.4 mM Glimepiride alone, 0.4 mM Metformin alone and Glimepiride + Metformin	Human breast cancer cell lines (CAL-148, MDA-MB-453, MDA-MB-231, MCF-7), CAL-148 xenografts	Suppressed tumor growth in CAL-148 xenografts. Induced G_1_/S phase cell cycle arrest and apoptosis. Activated AMPK, upregulated p53 and p21, downregulated cyclin D1 and CDK4.	[114]
Inhibitor	Gliclazide	6 mg/kg for 12 weeks	Colon cancer-inducing mouse	Reduced cell proliferation	[115]
Inhibitor	Nateglinide (NO_2_-NAT)	10~50 µM	Human pancreatic cancer cell lines (AsPC1, BxPC3)	Enhanced activity of caspases 3 and cell death.	[116]
Inhibitor	Repaglinide	1 µM to 200 µM	Neuroblastoma cells	Binds to FOXO3 DNA binding domain, silencing FOXO3’s transcriptional activity. Reduces cellular migration	[117]
